# The Origin and Functions of Exosomes in Cancer

**DOI:** 10.3389/fonc.2018.00066

**Published:** 2018-03-20

**Authors:** Chitra Rajagopal, K. B. Harikumar

**Affiliations:** ^1^Cancer Research Program, Rajiv Gandhi Centre for Biotechnology, Thiruvananthapuram, India

**Keywords:** exosomes, extracellular vesicles, cancer, angiogenesis, metastasis

## Abstract

Exosomes are nanovesicles having a maximum size of 150 nm and is a newly emerging focus in various fields of research. Its role in cargo trafficking along with its differential expression is associated with the disrupted homeostasis and provides an opportunity to defend against different diseases like cancer. Furthermore, exosomes are rich in cargos, which contain proteins and nucleic acids that directly reflect the metabolic state of the cells from which it originates. This review summarizes recent studies on tumor-derived exosomes with an overview about biogenesis, their functions and potential of using as diagnostic and prognostic markers. We also discussed the current challenges and microfluidic-based detection approaches that might improve the detection of exosomes in different settings. More intricate studies of the molecular mechanisms in angiogenesis, pre-metastatic niche formation, and metastasis can give more promising insights and novel strategies in oncotherapeutics.

## Introduction

**Figure 1:**
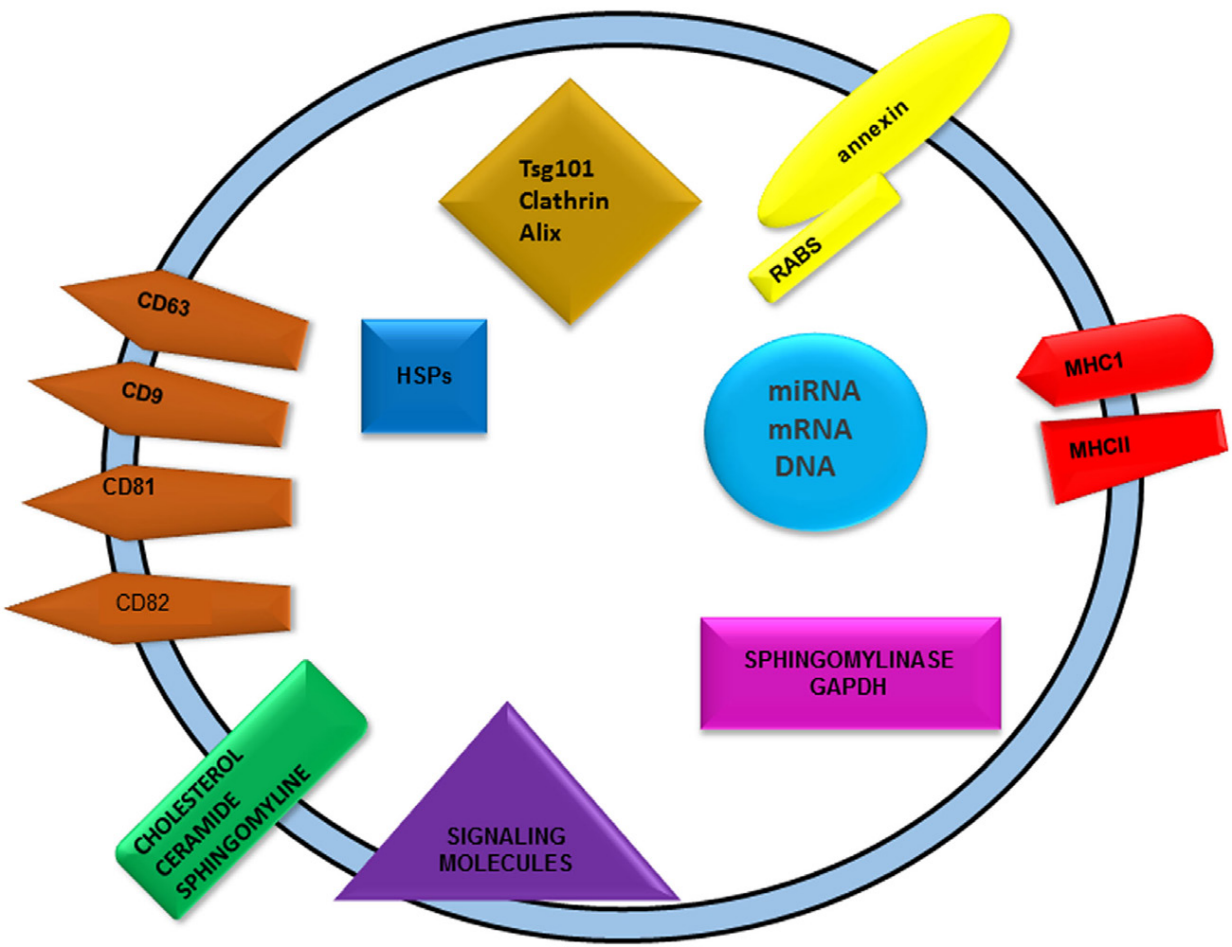
Graphical representation of exosomes showing general exosomal cargos. Nucleic acids, proteins, and lipids are the major cargos found in exosomes. Nucleic acid includes DNA, RNA along with non-coding RNAs like miRNAs. Different categories of proteins are present abundantly in exosomes, namely membrane and cytoplasmic proteins. Tetraspanins are the major membrane proteins such as CD9, 63, 81, 82, etc. Various heat shock proteins, alix, TSG101, and clathrin are cytoplasmic in its distribution. Presence of MHCI and II as transmembrane proteins indicates its role in immune cell induction. Sphingolipids such as ceramide and cholesterol are the major lipid species found in exosomes.

Exosomes are extracellular vesicles, which are functionally pleiotropic in nature. These nanoparticles have a size of 50–140 nm and carry specific cargos in it. They act as a transporting system for various biomolecules including DNA, RNA, proteins, and lipids. These are bilipid-layered vesicles that can carry their cargos on the plasma membrane and also in its cytoplasmic core (Figure 1). Exosomes were first identified in the process of elimination of transferrin (Tfr) receptors that occur during the maturation of reticulocytes. Reticulocytes undergo drastic cellular reprogramming in the initial stage of their maturation. Toward the last phase of this process, transferrin receptors are exocytosed with the aid of multivesicular bodies (MVBs) that carry 50-nm sized small vesicles, further named as exosomes (1). The release of exosomes occurred when MVBs fused with plasma membrane. MVBs are defined as intracellular endosomal organelles characterized by single outer membrane containing several internal vesicles. They are usually round or oval in shape and play a major role in several endocytic and trafficking functions (2).

Exosomes are found to be present in almost all kinds of body fluids such as blood, plasma, cerebrospinal fluid, bile, breast milk, etc. In normal homeostatic state, a basal level of exosomal release will aid in the elimination of cellular debris and also for cell-to-cell communication purposes. An increase in exosomal quantity and altered cargo expression can be considered as a potent biomarker for alteration of normal physiological states (1, 3, 4).

## Biogenesis of Exosomes

**Figure 2:**
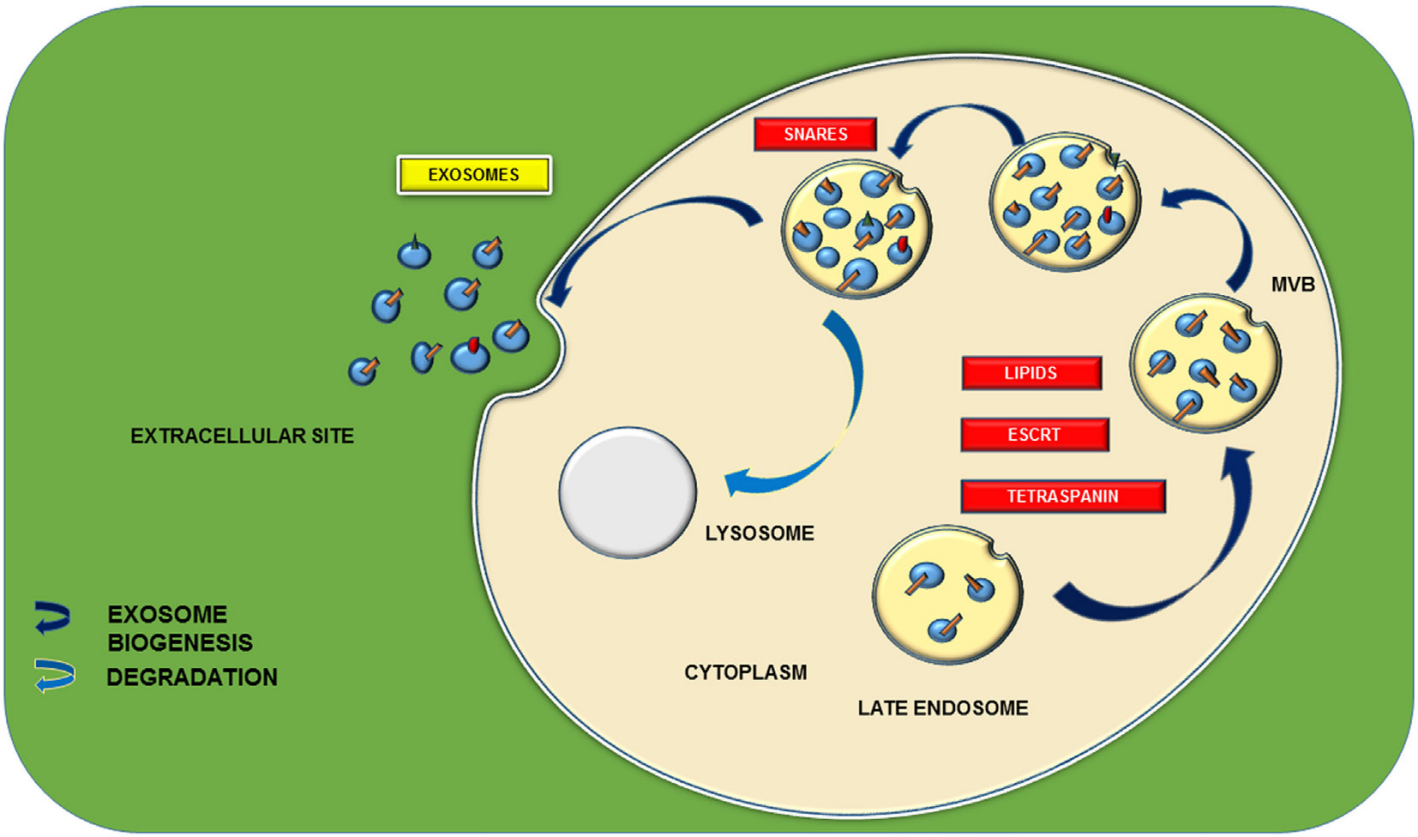
Schematic representation of exosomal biogenesis. Biogenesis of exosome is mainly through two types of pathways: endosomal sorting complex required for transport (ESCRT) dependent and ESCRT independent. ESCRT-dependent pathway is characterized with a set of proteins including ESCRT 0, I, II, and III and various tetraspanins, namely, CD9, 63, 81, 82, etc. They participate in the formation of multivesicular bodies (MVBs) from the late endosomes. ESCRT-independent pathway is proceeded by lipids such as ceramides and cholesterol. Further docking of these MVBs with the main plasma membrane with the aid of different soluble SNARE (*N*-ethylmaleimide-sensitive factor attachment protein receptors) complexes leads to the release of 40–150-nm sized nanovesicles.

Exosomes are surrounded by a lipid bilayer with a small fraction of cytosol and devoid of any kind of cellular organelles. Exosomes can be synthesized by means of two major pathways, and the process is highly regulated by multiple signal transduction cascades. Its release from the cell follows the normal exocytosis mechanism characterized with the vesicular docking and fusion with the aid of SNARE complexes (Figure 2). Mode of exosome biogenesis with details including the major proteins participating in the process is listed in Table 1.

**Table 1:** An account of mode of exosome biogenesis and proteins involved in the process of biogenesis.

Sl. No.	Mode of biogenesis	Proteins involved	Cell line used	Reference
1	Endosomal sorting complex required for transport (ESCRT) dependent	hsc73, annexin II, Gi2α	Murine DCs	(5)
2	ESCRT dependent	hsc70, alix	Rat erythrocytes	(6)
3	ESCRT dependent	CHMP4b, SKD1, alix	HeLa, HEK293 cells	(7)
4	ESCRT dependent	TSG101, VPS4b	DC2.4 cells	(8)
5	ESCRT dependent	Syndecan heparin sulfate, syntanin, alix	MCF-7 cells	(9)
6	ESCRT dependent	Hrs, STAM, TSG 101, VPS4	HeLa cells	(10)
7	ESCRT dependent	ESCRT, VPS34, VPS4	Mouse embryonic stem cells	(11)
8	ESCRT independent	Ceramide	Oli-neu cells	(12)
9	ESCRT independent	Rab27a, Rab27b	HeLa cells	(13)
10	ESCRT independent	Rab35, Rab11, TBC1D10A-C	Oli-neu cells	(14)
11	ESCRT independent	Sphingomyelinase 2	4T1 cells	(15)
12	ESCRT independent	Sphingomyelinase 2	GT1–7 cells	(16)
13	ESCRT independent	Sphingosine1 phosphate receptor	HeLa cells	(17)
14	ESCRT independent and Ca^2+^ dependent	Transferrin receptors	K562 cells	(18)
15	ESCRT independent and senescence associated	P53	LNCaP, 22Rv1, DU14 cells	(19)

### Endosomal Sorting Complex Required for Transport-Dependent Pathway

Endosomal sorting complex required for transport (ESCRT)-dependent pathway relays on a complex of various proteins as well as certain carbohydrate molecules for the successful biogenesis of exosomes through MVB formation. ESCRT consists of five distinct protein complexes, namely, ESCRT 0, I, II, and III, AAA ATPase, and Vps4. This process is initiated within the endosomal system. Molecular characterization of dendritic cell (DC) exosomes suggested its endosomal origin by indicating the presence of hsc73, an endosomal chaperone (5). In addition, chaperon-dependent exosomal cargo sorting is also reported by Geminard et al. Interaction of Tfr cytosolic domain with hsc70 is an important step in exosomal sorting of this particular receptor. Its further exit from reticulocyte is assisted by another protein called alix, which acts as an adaptor protein between hsc70 and Tfr receptor (6). Later in 2003, it was reported that two more proteins, CHMP4b (chromatin-modifying protein) and SKD1, cooperate with alix for the ESCRT-dependent exosomal biogenesis (7).

*In vitro* experiments conducted in Hrs-depleted cells revealed the significance of this particular protein in MVB formation. TSG101 and VPS4b (vacuolar protein sorting factor) are the two major ESCRT downstream proteins which were not found in Hrs-depleted DC exosomes and were enriched in control DCs (8). Baietti et al. conducted studies that unravel the importance of certain proteoglycans in exosomal synthesis and release. They reported the role of syndecan heparan sulfate proteoglycans and its adaptor syntanin in MVB formation. Motif-specific interaction of syntanin with the ESCRT downstream protein alix, which connects it with syndecan, causes intraluminal endosomal budding and abscission and leads to successful FGF–FGFR sorting (9). In 2013, Colombo et al. reported a study with RNA interference screen targeting, 23 ESCRT components and associated proteins in HeLa cells. This study reported ESCRT0 (Hrs, STAM1), ESCRT1 (TSG 101), and the late-acting VPS4 as the main genes responsible for exosomal biogenesis (10).

### ESCRT-Independent Pathway

Trajkovic et al. conducted studies to figure out the ESCRT-independent pathway of exosomal generation and found that ceramide has a significant role in MVB formation. The pathway was elucidated by tracking the PLP (proteolipid protein) trafficking. A comparative study of endosomal sorting of EGF with PLP revealed the decreased sorting of EGF with RNA interference in the expression of ESCRT and its associated proteins, which was just opposite in the case of PLP sorting. Study suggests the presence of certain microdomains in endosomal membrane that is rich in sphingolipids from which ceramide is formed whose cone-shaped structure is responsible for membrane curvature, which finally results in PLP sorting and vesicular abscission into the endosomes (12). Five RabGTPases were reported in the exosome biogenesis from HeLa cells in 2009. The study emphasizes on two Rabs, namely, Rab27a and Rab27b, which are elucidated as non-redundant in their functions. At the same time, Rab27b is pleotropic and involved in MVB size regulation and also in the transfer of these vesicles to the actin-rich cell cortex. Rab27a causes vesicular docking and its silencing can result in the formation of large multivesicular endosomes by means of the fusion of adjacent intra-endosomal vesicles (13). In 2010, Hsu et al. reported the significance of another isoform, Rab35, along with its regulation by a GTPase-activating protein TBC1D10A–C in exosomal docking and its biogenesis in the central nervous system. Knockdown of Rab35 along with Rab11 shows an increased accumulation of MVBs without its fusion and release (14). Experiments conducted in mouse 4T1 cells reveal the role of neutral sphingomyelinase 2 (nSMase2) enzymes in exosomal biosynthesis (15). Later in 2014, Guo et al. reported the significance of the same enzyme in exosomal packaging of prion protein. Knockdown of this particular enzyme in mouse model has shown the decreased release of exosomes from neuronal cells (16). These inferences emphasize on the importance of sphingolipids in exosomal cargo sorting and release.

Kajimoto et al. suggested the role of an inhibitory G-protein-coupled sphingosine-1-phosphate receptor in cargo sorting and exosome release. Constant supply of sphingosine-1-phosphate with the cytosolic localization of sphingosine kinase 2 enzyme to the receptor can cause continuous activation of the receptor and leads to cargo sorting and budding of intra-endosomal vesicles (17). The functional importance of calcium is already studied extensively in different cellular mechanisms. Senescence-associated increase in exosome release was also reported from prostate cancer (PCa) studies, which is actually mediated by p53 (19).

## Exosomal Cargos

Exosomes are bioactive vesicles that carry different molecular cargos. Proteins are one of the dominant contributors among them. Cells release exosomes in normal physiological conditions, which are mainly for cell-to-cell communication. Ligand molecules that are packaged in exosomes can be targeted to the adjacent cells, where they can interact with specific receptors (20). Exosomal cargo compositions could be considered as a prominent indicator of homeostatic aberrations in different body systems (21, 22). Exosomes can carry proteins, DNA, RNA including noncoding RNAs, and lipid species.

Both cytosolic and membrane bound proteins are present in exosomes, which are elucidated by different studies. Molecular characterization of DC exosomes revealed the presence of both the types of protein, among which hsc73 (hsp70 family member) is a predominant cytoplasmic protein that has a role in vesicular biogenesis. DC exosomal membrane is rich in tetraspanin proteins including CD9, 63, 81, and 82, which are responsible for their immunological functions (8). Recent analyses of urinary exosomes using mass spectrometry accounted for 49 different proteins that are mostly involved in carbohydrate and lipid metabolism. This gives a scope for exosome protein profiling in therapeutic field of metabolic disorders (23). Along with the abovementioned common exosomal markers, cells can sort specific proteins according to their physiological conditions. Cancerous cells can sort oncoproteins in exosomes, which can be incorporated into the adjacent bystander cells that finally lead to their neoplastic transition. Exosomes are immunologically active vesicles that can present antigens. DC exosomes carry both MHCI and II. But the actual way of immune mechanism is not yet elucidated (5). Skotland et al reported 107 lipid species from urinary exosomes of prostate cancer patients which include cholesterol, phospholipids and sphingomyelin (24). Sphingomyelin and ceramides indicates their association with exosomal biogenesis (12). In 2016, proteomic and lipidomic analyses of these vesicles revealed the presence of free fatty acid along with lysophosophatidyl derivatives as the positive curvature promoters and cardiolipin as the negative curvature promoter on exosomal membrane (25). The presence of 27-hydroxycholesterol (27-OHC) in breast cancer exosomes from ER^+^ breast cancer cell lines was also reported (26). 27-OHC is known to regulate the p53 expression and enhances the proliferation of ER^+^ breast cancer cells (27). Oncogenic cells produce miRNAs that modulate the expression of specific caretaker and tumor suppressor genes in adjacent cells, which promote further cancer progression and spreading. Most of the exosomal miRNAs are used as potent diagnostic markers. Interestingly mRNA content of exosomes was found to be different from that of its mother cell composition but miRNA composition of exosomes was similar (28). A detailed status of miRNA expression in exosomal vesicles in different cancer types is discussed in Table 2.

**Table 2:** Expression status of microRNAs as the exosomal cargo in different cancer types.

Type of cancer	Name of RNA cargo	Status of expression
Cervical cancer	mir-21, mir-146a	Overexpression (29)

Colorectal cancer	let7a, mir-21, mir-192, mir-221	Overexpression (30)

Hepatocellular cancer	mir-18a, mir-221, mir-222, mir-224	Overexpression (31)
	mir-101, mir-106b, mir-122, mir-195	Downregulation (31)

Lung cancer	mir-21, mir -21, mir-15, mir-200b-5p w, mir-200b-5p, mir-190b, mir-376a-5p, mir-378a, mir-379, mir-139-5p, mir-30a-3p, mir-629, mir-502-5p, mir-1974, mir-17, mir-100, mir-154-3p	Overexpression (32)
	mir-139-5p, mir-30a-3p, mir-378a	Downregulation (32)

Melanoma	mir-17, mir-19a, mir-21, mir-126, mir-149	Overexpression (33)

Ovarian cancer	mir-214, mir-140, mir-147, mir-135b, mir-205, mir-150, mir-149, mir-370, mir-206, mir-197, mir-634, mir-485-5p, mir-612, mir-608, mir-202, mir-373, mir-324-3p, mir-103, mir-593, mir-574, mir-483, mir-527, mir-603, mir-649, mir-18a, mir-595, mir-193b, mir-642, mir-557, mir-801, slet-7e, mir-21, mir-141, mir-200	Overexpression (34)

Prostate cancer	mir-409, mir-141	Overexpression (35, 36)

## Exosomes in Cancer

Although exosome release is a normal process, increase in its rate and its differential cargo expressions are favorable for oncogenic progression and metastases. Exosomes can be collected from blood, plasma, amniotic fluid, saliva, urine, etc, by ultracentrifugation and assessed for the molecular components such as DNA, RNA, miRNA, and proteins (37). Exosome-mediated transfer of different cargos promotes cancer progression and spreading. It can be explained by the bystander effect through coulee promoted infection. Exo-miRNA can communicate with the neighboring cells of the same tissue or the adjacent tissue *via* gap junctions or extracellular coulee. It can cause bystander effect, which make the cells as cancerous or can lead to its autophagy. Most of the studies are focusing on the increased release and bystander integration of these vesicles in response to radiation which is specifically named as radiation-induced bystander effect (RIBE) (38–40).

In esophageal squamous cell carcinoma, specific miRNAs have shown an increased expression with tumorigenesis and its aggressiveness. Exosomal miR-17, miR-19a, miR-21, miR-126, and miR-149 expression exhibited a positive correlation with the progression of metastatic sporadic melanoma by targeting around 40 genes (33). Exosomes isolated from the serum of PCa patients reported an overexpression of miR-141 (35). An increased expression of certain set of miRNAs including Let7a was also reported from the serum exosomal analysis of colorectal patients (20). Glioblastoma cells also exhibit miRNA upregulation (41). MiR-409 released from stromal fibroblast cells were involved in human prostate tumorigenesis by inducing EMT and downregulating the expression of tumor suppressor genes (RSU1 and STAG2) (36).

Oncogenic proteins enclosed in exosomes can lead to spreading of tumor to the adjacent tissues. Recently, a novel protein, myoferlin is reported to be present in pancreatic and breast cancer cells, described as a common protein in cancer cell releasing exosomes. The study unravels the importance of myoferlin in cancer progression and metastases with increased exosomal biogenesis and packaging of nucleic acid cargo to adjacent cells (42). The oncogenic receptor EGFRvIII was shown to be transported between glioma cells with the aid of exosomes resulted in the transfer of oncogenic activity, transforming phenotype and EGFRvIII-dependent transcription (43). Proteomic analysis of urinary exosomes identified 49 proteins that were considered as significant. Interestingly, functional and biological interpretations of the proteins revealed that they have diverse functions but none related to reproductive functions. This further explained that outer part of urinary exosomes chiefly contributes to various biological functions attributed to exosomes (23). A comparison of different exosomal studies reveal that during the course of progression of cancer, it shows differential sorting of exosomal content. Release of certain proteins will be higher during the onset of cancer, but toward the phase of metastases, a decrease in its expression can be found with the increased release of other proteins. Cancer onset related exosomal markers differ from that of the metastases related exosomal markers such as integrins and tetraspanins (44). DNA fragments are also found associated with exosomes including full-length H-ras and sequences of N-ras oncogenes were reported as the exosomal contents, released by mouse brain tumor cells (45).

## Exosomes and Tumor Microenvironment

Tumor microenvironment consists of cellular and acellular factors contributed by the tumor and its surroundings. Its main composition includes extracellular matrix (ECM), cancer-associated fibroblasts (CAFs), inflammatory immune cells and tumor-associated vasculature. CAFs play a major role in the maintenance of tumor microenvironment with the release of different proteins to the extracellular sites that are involved in various signal transduction pathways and their regulations. Almost all these factors are released with the aid of exosomes (46). Inflammatory immune cells are one of the major contributors in the tumor microenvironment. Exosomes can maintain this microenvironment by immune system activation. Most of the studies focus on DC-derived exosomes. Molecular characterization of DC-derived exosomal studies conducted in 1999 unravel exosome-mediated immunological induction in tumor progression. These vesicles may interact with T cells directly or indirectly with the aid of antigen-presenting cells (APCs) and can trigger immune responses. Proteomic data show the presence of class 1 and 2 MHCs on exosomal membrane and can present antigens to induce T cells. Tetraspanin membrane proteins are also reported as DC exosomal proteins that can trigger immune system. Presence of a membrane protein MAC1 (a β2 integrin also known as the type 3 complement receptor, CR3) also suggests the possibilities of dendritic exosomal interactions with different immune cells such as lymphocytes. Detailed studies revealed a major fact that exosomes cannot interact with T cells directly; they are mostly involved in the sensitization of other DCs, which is concluded with the report of increased rate of exosomal release from the immature DCs (5). Antigen-presenting capacity of exosomes is elucidated with experiments in murine DCs. Hrs-depleted DCs have shown decreased MHC expression which is opposite in the case of control DCs. Absence of ESCRT pathway downstream proteins decreases MHC expression, this is an indication of active sorting of MHC molecules in dendritic exosomes which can possibly make significant immunological triggers (8).

Fc receptor-mediated recognition of exosomes by macrophages promotes tumor growth and metastasis. These vesicles with functional proteins induce the immune system with the activation of macrophages, accomplished with the cytoskeleton rearrangements of the cell (11). Conditioning of tumor microenvironment with the modulation of cytoskeleton-associated protein has proved in PCa also (47). Increased expression of pro-inflammatory markers on macrophages in response to exosomes derived from gastric cancer cells is an evidence of tumor-promoting inflammatory cellular environment. Enhanced NF-κB activation that is already established as the key molecule involved in inflammatory responses is also reported from the above study (48). One of the major cellular elements of tumor microenvironment is stromal fibroblast cells. Exosome-mediated horizontal gene transfer between cancer cells and the stromal cells contribute a lot to the maintenance of cancer favorable inflammatory environment. Increased release of unshielded exosomal RN7SL1, RNA acts as the ligand for pattern recognition receptors from stromal fibroblast cells, can promote aggressive cancer progression with increased inflammatory responses (49). Exosome-mediated metabolic reprogramming of cancer cells by herpes viruses results in the development of tumor microenvironment. The transfer of virus-encoded miRNA to the cancer cells leads to the shift toward aerobic glycolysis and promotes the growth of the infected cell thereby increase the fitness of the virus (50).

On the other hand, immune suppression is a major trait of tumor microenvironment. Cancer cells show different strategies to inhibit the immune system action for the clearance of tumor deposition. Recent studies revealed that exosomes are potent players in this process. Hypoxic tumor-derived microvesicles can block NK cells, well known for tumor surveillance. These vesicles are loaded with miR-23a which is absent in vesicles from normoxic cancer cells. This emphasizes hypoxia as a prominent characteristic for an immunocompromised tumor microenvironment (51). Hypoxic PCa exosomes can be responsible for the enhanced invasiveness, stemness, and microenvironment changes in PCa cells. Further exosomes secreted by PCa cells under hypoxic conditions cause neoplastic transition of fibroblasts in tumor microenvironment (52).

## Exosomes in Angiogenesis

Angiogenesis is a major process which regulates nutrient availability of fast growing solid tumors. Exosomal interaction and uptake of endothelial cells (ECs) will induce angiogenesis with the incorporation of vesicular cargos such as tetraspanin 8 and CD106 and 49d, which can activate vascular endothelial growth factors (VEGFs). VEGFs can cause EC proliferation, migration, sprouting, and maturation of EC progenitors (53). Exosomes released from the endothelial progenitor cells also interact with mature ECs and its cargo integration triggers AKT signaling, resulting in angiogenesis (54). Hypoxia is a major factor that induces angiogenesis. Exosomes also play a major role in the communication between hypoxic tumors and its microenvironment. Alterations in molecular constituents of exosomes during hypoxic conditions will induce the EC proliferation and tube formation. *In vitro* studies of tube formation in normoxic and hypoxic human umbilical vein endothelial cells (HUVECs) that were incubated with exosomes released from human leukemic cells have suggested differential cargo expressions in hypoxic vesicles and its role in angiogenesis (55). Beyond endothelial cell-to-cell interaction, communication between metastatic tumor cells and ECs can activate various cytoskeletal proteins such as RAC1, which induce angiogenesis (56). Role of melanoma exosomes in the regulation of endothelial tubular morphology by inducing tubular sprouting and spheroid formation was also reported (57). Tetraspanins were discussed for its role in exosome biogenesis, cargo sorting, cancer progression, etc. Studies suggest that tetraspanins are key players in the process of angiogenesis (58). Expression of genes related to vascular remodeling, such as ephrin A3 and PTP1B, were also reported in response to the exosomal miR-210 that is involved in the formation of blood vessels in hypoxic tumor tissues (59).

## Exosomes and Metastasis

**Figure 3:**
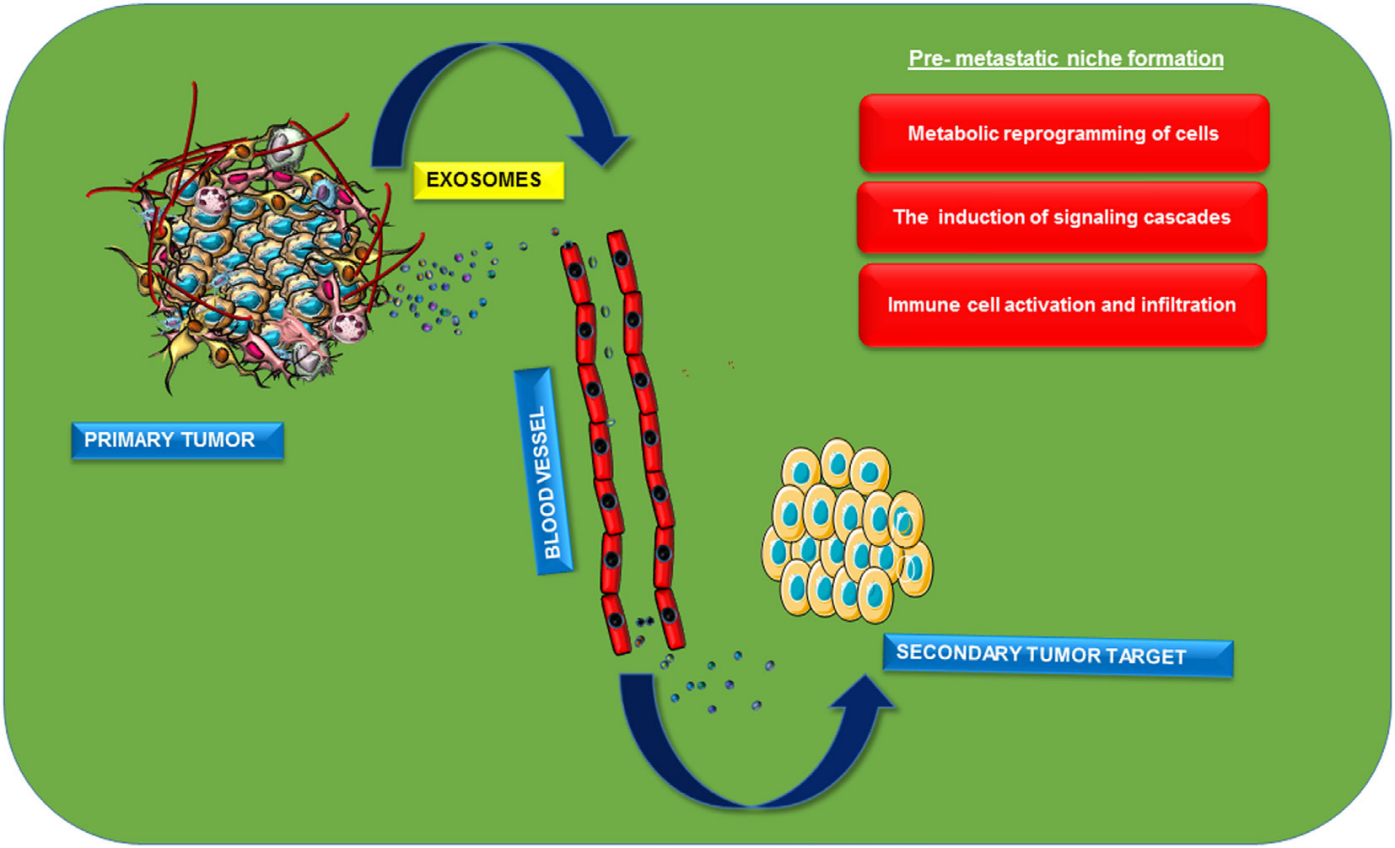
Role of exosomes in pre-metastatic niche formation. Exosomes will be released from the primary cancer cells into the extracellular sites. Distribution and specific organotropic integration of these vesicles with oncoproteins or nucleic acids as cargos lead to the development of pre-metastatic niche in the secondary site of cancer metastasis. Induction of different signaling pathways and activation of different immune cells in the secondary site helps in the maintenance of cancer favorable inflammatory microenvironment that promotes successful cancer cell metastasis.

Metastasis is the process of detachment and successful invasion of cancerous cells from the primary site of tumor to the secondary one mainly through blood flow. It is mainly characterized by different steps including metabolic reprogramming of cells, loss of cell connections with increased action of matrix metalloproteases, and diapedesis of cancerous cells and its integration to specific target sites. Metastasis is the process that makes the disease more dreadful with its recurrence (60). The process of epithelial mesenchymal transition (EMT) is also a major contributor for metastasis (61). EMT is characterized by the transformation of tightly packed epithelial cells to loose motile mesenchymal cells, afterward they move freely through blood or by other means. This complex process is characterized by altered expression patterns of transcription factors including Snail, Twist, etc, which are mainly regulated at the transcriptional, translational, and post-translational levels. This is actually preceded by the complex interplay between various signaling molecules, among which one of the major one is TGF-β (62). Recent studies have suggested possible roles of exosomes in the process of metastasis. Early metastatic expression of exosomal miR-105 is reported in the transformation of non-metastatic cells to metastatic cells. Further suppression of miR-105 expression restores vascular integrity and inhibits the process of metastasis in breast cancer. This miRNA targets ZO-1a, tight junction protein, and observed an inverse correlation between the expression of miR-105 and ZO-1 (60). The study also suggested the possible role of miR-105 as a non-invasive marker for the prediction or early diagnosis of breast cancer metastasis (60). Role of exosomes in metastatic cell invasion can be explained by seed and soil theory in a different perspective. Primary metastatic cells can send oncogenic biomolecules to the target site before cell invasion itself. This will architect a pre-metastatic niche in the target organ that leads to the successful metastasis of these cells (Figure 3). Exosomal release of CD97 plays a critical role in pre-metastatic niche formation in gastric cancer cells (63). Uptake of exosomes released from stromal cells by breast cancer cells and vice versa suggested the role of these nanovesicles in metastases (64). Pre-metastatic niche will be mainly programmed by the altered expression of various signal transduction pathways. Macrophage migratory inhibitory factors packaged in pancreatic ductal adenocarcinoma exosomes will be taken up by Kupffer cells that can secret TGF-β, leads to the increased expression of fibronectin in hepatic stellate cells which finally cause the infiltration of macrophages to liver (65). Exosomal miR-21 and 29a can act as ligands for toll-like receptors and can induce inflammatory responses in pre-metastatic niche formation (55). Pre-metastatic niche formation contributes to the organotropic metastasis as reported by the studies with hematopoietic bone marrow cells. These progenitors have shown an increased expression of VEGF-1 in target sites and formed cellular clusters before cancer cells have been invaded and induced an overexpression of fibronectin in resident fibroblast cells. Thereby the secondary site will be favorable for the easy invasion of the primary cancer cells (66). Integrins are transmembrane receptors involved in adhesion, migration, cell differentiation, etc. Increased expression of these proteins in late stages of cancer is considered as an indication of metastasis. Invasive integrin positive PCa cells can package these transmembrane receptors in exosomes and will be targeted to the integrin negative cells and there they can promote cell invasion (67). Organotropic metastases promoted by the expression of different isoforms of integrins in various tissues were also reported (68). A comparative proteomic characterization of metastatic and non-metastatic cellular exosomes accounted the differential expression of proteins between the samples. Vimentin, hepatoma-derived growth factors on exosomal membrane and casein kinase II, and α annexin-like molecules in its cytoplasmic lumen were reported from murine metastatic bladder cell lines (69). Exosome-packaged MET oncogenes will have the potency to promote cellular transformation, tumor cell proliferation, survival, motility, invasion, and metastasis (70). Proteomic analysis of exosomes released from A431 cells revealed the presence of various signaling molecules related to cellular movements including integrin and tetraspanin (71). Exosomal hsp90 along with annexin-II released to the extracellular sites have important role in the activation of plasminogen, its conversion into plasmin will degrade ECM and cell adhesion junctions between adjacent cells and promote EMT (72). MiR-409, packaged in extracellular vesicles, was reported to promote metastatic progression in human PCa (36). nSMase2, which mediates miRNA sorting in to exosomes, can be induced by hypoxia leading to the secretion of angiogenic miRNAs, such as miR-210, to facilitate tumor angiogenesis and thereby promote metastasis (15). MiRNAs from astrocytes have the potency for epigenetic regulation of PTEN mRNA and protein. Astrocyte-derived exosomes are found to mediate trafficking of PTEN-targeting miRNAs to metastatic tumor cells and promote the process of metastasis (73). Gastric cancerous cells produce Let-7-containing exosomes and may have oncogenic metastatic activity which is contradictory to its normal tumor suppressor activity (74). Tumor microenvironment is playing a major role in metastasis. It is reported that exo-annexin II released from breast, lung, and brain cancer cells can promote metastases by triggering macrophage-mediated activation of p38MAPK, NF-κB, and STAT3 pathways and increased secretion of IL-6 and TNF-α in tumor microenvironment (75). Autocrine induction of Wnt-planar cell polarity (PCP) signaling in stromal fibroblast cells in breast cancer can maintain a metastatic microenvironment, which will also promote cell invasion (46). Exosomal recruitment of CXCR4 expressing stromal cells leads to metastatic microenvironment formation and promotes liver metastasis of colorectal cancer cells (76). Hypoxic studies in cancer cells proved differential expression pattern of proteins in hypoxic tumor exosomes and their importance in cell stemness and metastasis. A comparative study of hypoxic and normoxic prostate exosomes accounted for higher metalloprotease activity and increased levels of diverse signaling molecules such as TGF-β2, TNF-α, IL-6, TSG101, Akt, ILK1, and β-catenin (52). Repeated secretomic studies accounted for more proteins including metastatic markers (MET, S100A8, S100A9, TNC), signal transduction molecules (EFNB2, JAG1, SRC, TNIK), lipid rafts, and lipid raft-associated components (CAV1, FLOT1, FLOT2, PROM1) in exosomes derived from metastatic SW620 cells (77).

## Exosomes as Diagnostic Agents or in Cancer Therapeutics

Cancer is one of the major diseases which show a high rate of resistance toward various therapeutic strategies. Recent studies suggest exosomes as major players in increased survival rate of cancerous cells after chemotherapy. Drug-resistant breast cancer cells transfer the resistive power to the adjacent sensitive cells through exosomes. Horizontal transfer of specific cargos with the aid of these biological nanovesicles alters specific gene expression patterns in recipient cells (78). Several potential biomarkers were identified through proteomic analyses of cancer-derived exosomes from various types of cancers (23, 28, 41). Various studies in different cancer subtypes showed that exosomes can be considered as potential biomarkers owing to its ubiquitous presence in different body fluids.

Rabinowits et al. reported that in lung adenocarcinoma, circulating total miRNAs can be used as a screening tool because they observed a significant difference between patients and healthy controls (79). Several miRNAs (miR-17-5p, miR-21, miR-550, miR-10b) were characterized from exosomes isolated from peripheral blood of pancreatic cancer patients, suggesting the use as early biomarkers (80). Similarly, miR-373 was significantly elevated in the serum of TNBC patients as compared to control subjects (81). A panel of 16 miRNAs was abundant in serum exosomes isolated from CRC patients (82), and Matsumura et al. showed that miRNAs overexpressed in CRC tissue samples were mostly converged into miR-17–92 cluster. They also showed that circulating levels of exosomal miR-19a was a marker which can be used to assess both the overall and disease-free survival (83). LINC00152, AK001058, INHBA-AS1, MIR4435-2HG, UCA1, and CEBPA-AS1 were the long non-coding RNAs found in exosomes, and it was shown that they can be used as a blood-based biomarker in gastric cancer (84, 85). Caludin-4 (a tight junction protein) containing exosomes released from ovarian cancer cells was found in the peripheral blood system, and studies suggested that developing sensitive assays for determining caludin-4 levels can be a screening criteria for ovarian cancer (86). Pre-clinical studies confirmed that MIF present in exosomes can predict the liver metastasis (65). Yet another study reported the potential diagnostic use of glypican-1 (GPC1), a cell surface proteoglycan which was significantly elevated in exosomes of pancreatic cancer patients. When we analyzed the published literature on cancer-derived exosomes, it was observed that majority of studies were focused on cancer progression and made attempt to establish its usefulness to predict prognosis.

However, the potency of these vesicles in the field of disease diagnosis and therapeutics are entangled in fundamental technical challenges. The major hurdle is its isolation and the chance of contamination with other extracellular vesicles which is followed by the limited life span of its surface markers once it is out of the living system. Conventional strategy for the isolation of exosomes is differential centrifugation including ultracentrifugation at 100,000 *g* followed by sucrose gradient centrifugation as a purification step. Inconsistent results of this practice further lead to the development of different exosome isolation kits (87). An account of commercially available exosome kits is listed in Table 3. But research in the field of nano-microfluidics provides more promising alternatives instead of conventional methods (88). With advanced technologies, there is a strong interest in developing the rapid and high-throughput platform for exosome-based diagnostics in clinical settings without the purification of exosomes. Ex-Chip is one such approach and is a microfluidic device that captures and stains exosomes with CD63 antibody and a fluorescent dye (89). In ExoScreen system, exosomes were captured by two types of antibodies (CD9 and 147) and are detected by photosensitizing beads (90). ExoSearch, yet another microfluidic device, allows enriched preparation of blood plasma exosomes for *in situ*, multiplexed detection using immunomagnetic beads (91). Furthermore, the authors demonstrated the potential of ExoSearch in ovarian cancer using three plasma exosomal markers (CA-125, EpCAM, and CD24) in diagnostics.

**Table 3:** List of major exosome isolation kits including name of the kit, principle of action, and final state of yield.

No.	Name of the kit	Catalog no.	Principle of the kit	Final product
1	Capturem (clontech)	635723	Isolation with exosome interacting non-antibody molecules	As elute

2	ExoQuick	EXOQ20A-1	Precipitation	As pellet

3	Miltenyi	130-110-913	Pulling out the exosomes with immunolabelled magnetic beads	As elute

4	MiRCURY (Qiagen)	76743	Precipitation	As pellet

5	PureExo (Bio)	P100	Precipitation	As pellet

6	ThermoFisher	4478359	Precipitation	As pellet

At the same time, exosomes can be used for drug targeting. Researchers are trying to use exosomes in packaging of drugs instead of using synthetic nanoparticles. Exosomes are promising agents for drug delivery with its properties such as low immunogenicity, innate stability, and high delivery efficiency. Peptide-conjugated exosomes loaded with curcumin as a drug has been proved as a good system for the effective drug delivery for brain ischemia (92). This strategy can reduce the rate of loss of drug in blood stream with its tissue specific targeting. Exosome-mediated intranasal administration of catalase has been proved for its decreased protease degradation and sustained release into the brain for Parkinson’s disease (93). Natural polyphenols are established against different forms of cancer. But its decreased bioavailability and stability is found to be as a major issue in this therapeutic field. Treatment of mesenchymal stromal cells with paclitaxel, a drug for cancer, leads to its uptake and release in exosomes proclaim the scope of more studies in this field (94). Ovarian xenograft studies using A2780 cells revealed the increased anticancer activity of anthocyanin than that of the conventional drugs (paclitaxel) (95). As we discussed in the Section “Introduction,” all the body fluids have exosomes. Most of the drug delivery works are done with the vesicles isolated from the bovine milk. It is considered as a good exosomal source for the drug delivery purposes because of no adverse immune and inflammatory effects (96). The ongoing and completed clinical trials using exosomes as diagnostic or therapeutic agents in cancer are listed in Table 4.

**Table 4:** Ongoing and completed clinical trials involving exosomes as therapeutics or diagnostic agents [Source: National Institute of Health (NIH) clinical trial registry].

S.No	Title of the study	Type of cancer	Study design	Starting date	NCT number
1	Interrogation of exosome-mediated intercellular signaling in patients with pancreatic cancer	Pancreatic cancer	Prospective trial: observational	March 2015	NCT02393703

2	Circulating exosome RNA in lung metastases of primary high-grade osteosarcoma	Osteosarcoma	Prospective trial: observational	May 2017	NCT03108677

3	ncRNAs in exosomes of cholangiocarcinoma	Cholangio carcinoma	Prospective translational study with preclinical and clinical phases	May 2017	NCT03102268

4	Edible plant exosome ability to prevent oral mucositis associated with chemoradiation treatment of head and neck cancer	Head and neck cancer	Intervention model: parallel assignment	August 2012	NCT01668849

5	Exosome testing as a screening modality for human papillomavirus-positive oropharyngeal squamous cell carcinoma	Oropharyngeal squamous cell carcinoma	Observational, single-institution pilot/feasibility study	February 2015	NCT02147418

6	Diagnostic accuracy of circulating tumor cells (CTCs) and onco-exosome quantification in the diagnosis of pancreatic cancer – PANC-CTC (PANC-CTC)	Pancreatic cancer	Prospective trial: observational	February 2017	NCT03032913

7	Metformin hydrochloride in affecting cytokines and exosomes in patients with head and neck cancer	Head and neck cancer	Intervention model: parallel assignment	March 2017	NCT03109873

8	Study of molecular mechanisms implicated in the pathogenesis of melanoma. Role of Exosomes (EXOSOMES)	Melanoma	Intervention model: single Group assignment	December 2014	NCT02310451

9	Olmutinib trial in T790M (+) NSCLC patients detected by liquid biopsy using BALF extracellular vesicular DNA	NSCLC	Intervention model: single Group assignment	July 2017	NCT03228277

10	Study investigating the ability of plant exosomes to deliver curcumin to normal and colon cancer tissue	Colon cancer	Intervention model: factorial assignment	January 2011	NCT01294072

11	Effect of plasma derived exosomes on cutaneous wound healing	Ulcer	Intervention model: single group assignment	September 2015	NCT02565264

12	Circulating exosomes as potential prognostic and predictive biomarkers in advanced gastric cancer patients (“EXO-PPP Study”)	Gastric cancer	Observational model: case control	January 2013	NCT01779583

13	Clinical research for the consistency analysis of PD-L1 in cancer tissue and plasma exosome (RadImm01)	Non-small cell lung cancer (NSCLC)	Intervention model: single group assignment	October 2016	NCT02890849

14	Clinical validation of a urinary exosome gene signature in men presenting for suspicion of prostate cancer	Prostate cancer	Observational model: cohort	May 2014	NCT02702856

15	Trial of a vaccination with tumor antigen-loaded dendritic cell-derived exosomes (CSET 1437)	Unresectable non-small cell lung cancer responding to induction chemotherapy	Intervention model: single Group assignment, Phase II trial	December 2009	NCT01159288

16	Clinical research for the consistency analysis of PD-L1 in lung cancer tissue and plasma exosome before and after radiotherapy (RadImm02)	Lung cancer	Intervention model: single Group assignment	October 2016	NCT02869685

17	Anaplastic thyroid cancer and follicular thyroid cancer-derived exosomal analysis *via* treatment of lovastatin and vildagliptin and pilot prognostic study *via* urine exosomal biological markers in thyroid cancer patients	Thyroid cancer	Observational model: cohort	August 2016	NCT02862470

A couple of studies were reported on the importance of exosomes in vaccine development. Serum-derived exosomes from pigs are revealed for its use for the development of the vaccine against porcine reproductive and respiratory virus (PRRSV) (97). Studies conducted with the exosomes isolated from advanced stage of T-cell lymphoma have proved its role in triggering the immune response (98). It is found that exosomes released from glioma cells have the potency to act as antigen sources for immune system activation (99). Experiments conducted with dendritic exosomes revealed its anti-tumor activity by the induction of T-lymphocytes (100). Anti-cancer immunity of DC-derived exosomes has also proved in B16F10 melanoma model (101). It gives the scope of exosomes in developing vaccines against cancer, one of the dreadful diseases to tackle.

## Future Perspectives on Cancer Exosomes

Exosomes are considered as heterogeneous entities, and the complexity of exosomes is still not thoroughly understood. We propose following areas which require an urgent attention to understand the complexity of exosomes.

1. More sophisticated techniques and methodologies for isolating cancer exosomes.
2. The nature of the cargo in the exosomes majorly depends on the origin of the cells from where the exosomes get released. Therefore, how the cargo is packed in exosomes needs to be fully elucidated. It is important because cancer cells are known for their heterogeneity and nature of cargo from each cancer cell will be different. This will further help us in designing strategies for early diagnosis and monitoring response to treatment using exosomes.
3. The role of exosomes in diagnostics and therapeutics is mostly confirmed using cancer cell lines and animal models. A large-scale, randomized clinical trials must be conducted in different types of cancers for further validation and establishing the use of exosomes as point of care diagnostics.
4. Identification of exosomes based biomarkers in response to conventional cancer therapies to study the treatment response.
5. The potential use of exosomes as delivery vector needs more critical evaluation. How tractability can be improved? Whether multiple drugs can be packaged in exosomes? As we are in the era of personalized medicine, whether we can make personalized approach for delivering therapeutically relevant exosomes?
6. Till date, there is a dearth of well-defined guidelines for manufacturing, storage and administration of therapeutically relevant exosomes. There is an urgent need to develop guidelines with respect to safety and quality of exosomes and GMP standards to be followed.

## Conclusion

These studies on the composition and biogenesis of exosomes provide us with detailed insight into the role of cancer-derived exosomes in tumor growth, drug resistance, metastasis, and angiogenesis. The exosomes are rich in different types of cargo and reflect the state of tumor cells from which it is derived and can be explored as minimally invasive biomarkers for early detection, diagnosis and prognosis of different type of cancers.

## Author Contributions

All authors listed have made substantial and intellectual contribution in the preparation of the manuscript and final version was approved for publication.

## Conflict of Interest Statement

The authors declare that the research was conducted in the absence of any commercial or financial relationships that could be construed as a potential conflict of interest.
